# Whole-Genome Draft Sequences of Three Multidrug-Resistant *Klebsiella pneumoniae* Strains Available from the American Type Culture Collection

**DOI:** 10.1128/genomeA.00312-13

**Published:** 2013-05-30

**Authors:** Christopher A. Broberg, Michelle Palacios, Virginia L. Miller

**Affiliations:** Department of Genetics, University of North Carolina at Chapel Hill, Chapel Hill, North Carolina, USAa; Department of Microbiology and Immunology, University of North Carolina at Chapel Hill, Chapel Hill, North Carolina, USAb

## Abstract

Infection with multidrug-resistant *Klebsiella pneumoniae* is a significant problem worldwide, requiring a better understanding of how various strains are able to defeat current antibiotic therapies and cause disease. Here, we report the draft genome sequences of three *K. pneumoniae* strains harboring the SHV-18, KPC-2, or NDM-1 β-lactamases.

## GENOME ANNOUNCEMENT

*Klebsiella pneumoniae* is a Gram-negative rod of the *Enterobacteriaceae* family and is a frequent cause of urinary tract infections, pneumonia, liver abscess, and burn wound infections in community and clinical settings (1). It recently has gained attention for the appearance of multiple drug-resistant strains in the clinic, as well as high mortality rates associated with septic infection (2). The emergence of pan-resistant *K. pneumoniae* strains in the clinic may herald the end of our ability to readily treat Gram-negative bacterial infections (3, 4).

Despite its clinical significance, little work has been done to date to characterize *K. pneumoniae* virulence. An in-depth analysis of how *K. pneumoniae* causes disease, as well as a complete understanding of its mechanisms of antibiotic resistance, is necessary to prevent the spread of this pathogen in the clinic as well as the transfer of genetic elements containing antibiotic resistance genes to other bacterial species.

To gain a better understanding of *K. pneumoniae* pathogenesis, we sequenced three strains that represent different classes of antibiotic resistance. Strain 700603 was the first *K. pneumoniae* strain identified that carries the SHV-18 extended-spectrum β-lactamase (ESBL), capable of hydrolyzing oxyimino-β-lactams in addition to penicillins and narrow-spectrum cephalosporins (5, 6). BAA-1705 contains the *Klebsiella pneumoniae* carbapenemase KPC-2, which has activity against cephamycins and carbapenems in addition to the substrate range of ESBLs (5). BAA-2146 encodes the New Delhi metallo-β-lactamase NDM-1 that confers resistance to all β-lactam antibiotics except aztreonam (7). BAA-1705 and BAA-2146 are recommended as controls for the detection of KPC-2 and NDM-1 in PCRs to confirm the presence of these β-lactamases in clinical isolates of *Klebsiella* and other Gram-negative species (8). All three strains are available from the American Type Culture Collection (ATCC).

Sequencing was performed at the University of North Carolina High Throughput Sequencing Facility (UNC HTSF) using an Illumina HiSeq 2000 instrument generating 2 × 100-bp paired-end reads. Paired reads (56,096,066, 55,407,574, and 58,438,668 reads) were obtained for a total of 5.00, 4.98, and 5.25 gigabases for 700603, BAA-1705, and BAA-2146, respectively.

*De novo* assemblies were generated using the CLC Genomic Workbench v. 5.5.1. The 700603 assembly resulted in 103 contigs with a G+C content of 57.8%, for a total of 5,461,663 bp and 5,313 coding sequences. The assembly of BAA-1705 yielded 169 contigs with a G+C content of 57.1%, for a total of 5,662,914 bp and 5,660 coding sequences. The BAA-2146 assembly resulted in 109 contigs with a G+C content of 57.0%, for a total of 5,680,367 bp and 5,636 coding sequences. Contigs were annotated using the Prokaryotic Genomes Automatic Annotation Pipeline (PGAAP) through NCBI.

### Nucleotide sequence accession numbers.

These whole-genome shotgun projects have been deposited at GenBank under the accession numbers AOGO00000000, AOGQ00000000, and AOCV00000000. The versions described in this paper are the first versions, AOGO01000000, AOGQ01000000, and AOCV01000000 for strains 700603, BAA-1705, and BAA-2146, respectively.
